# Virtual Education Program to Support Providers Caring for People With Intellectual and Developmental Disabilities During the COVID-19 Pandemic: Rapid Development and Evaluation Study

**DOI:** 10.2196/28933

**Published:** 2021-10-07

**Authors:** Anupam Thakur, Cheryl Pereira, Jenny Hardy, Nicole Bobbette, Sanjeev Sockalingam, Yona Lunsky

**Affiliations:** 1 Centre for Addiction and Mental Health Department of Psychiatry University of Toronto Toronto, ON Canada; 2 School of Rehabilitation Therapy Queen's University Kingston, ON Canada

**Keywords:** COVID-19, coronavirus, pandemic, intellectual disability, mental health, community of practice, ECHO, virtual, capacity-building

## Abstract

**Background:**

People with intellectual and developmental disabilities are at increased health-related risk due to the COVID-19 pandemic. Virtual training programs that support providers in caring for the physical and mental health needs of this population, as well provide psychological support to the providers themselves, are needed during the pandemic.

**Objective:**

This paper describes the design, delivery, and evaluation of a virtual educational COVID-19–focused Extension for Community Healthcare Outcomes program to support providers during the COVID-19 pandemic in caring for the mental health of people with intellectual and developmental disabilities.

**Methods:**

A rapid design thinking approach was used to develop a 6-session program that incorporates mindfulness practice, a wellness check, COVID-19–related research and policy updates, a didactic presentation on a combination mental health and COVID-19 related topic, and a case-based discussion to encourage practical learning. We used the first 5 outcome levels of Moore’s evaluation framework—focusing on participation, satisfaction, learning, self-efficacy, and change in practice—which were rated (out of 5) by care providers from health and disability service sectors, as well as additional reflection measures about innovations to the program. Qualitative feedback from open-text responses from participants were analyzed using modified manifest content analysis.

**Results:**

A total of 104 care providers from health and disability service sectors participated in the program. High levels of engagement (81 participants per session on average) and satisfaction (overall satisfaction score: mean 4.31, SD 0.17) were observed. Self-efficacy (score improvement: 19.8%), support, and coping improved. Participants also rated the newly developed COVID-19 program and its innovative components highly. Open text feedback showed participants felt that the Extension for Community Healthcare Outcomes program expanded their knowledge and competency and created a sense of being part of a community of practice; provided value for the COVID-19 innovations; supported resource-sharing within and beyond program participants; and facilitated changes to participants’ approaches to client care in practice and increased participants’ confidence in supporting clients and families.

**Conclusions:**

The Extension for Community Healthcare Outcomes program is an effective model for capacity-building programs with a shared-learning approach. Future iterations should include targeted evaluation of long-term outcomes such as staff burnout.

## Introduction

Countries across the world have responded to the COVID-19 pandemic with rapid deployment of public health measures and hospital-based care for the acutely unwell. Various population groups, including people with intellectual and developmental disabilities, are marginalized and underserved in the health care system, both during and prior to COVID-19 [1]. They are at increased risk for poorer outcomes since the start of the COVID-19 pandemic, which includes a higher risk of COVID-19 infection, severe complications, and mortality [1-5]. The COVID-19 pandemic presents twin challenges of (1) addressing clinical vulnerabilities experienced by people with intellectual and developmental disabilities [4], and (2) responding swiftly to ever-evolving information. Social service providers and health care providers play a major role in caring for this marginalized group in the community [6,7]. Effective training programs for health and community service providers are needed because of the increased vulnerability to risk of infections of people with intellectual and developmental disabilities, as well as a lack of information available about supporting the needs of people with intellectual and developmental disabilities.

Experiences from previous pandemics suggest the need to support health care workers by increasing their mental health awareness and encouraging self-care [8]. Health care providers and support staff in the community are facing psychological distress during the COVID-19 pandemic. This has been attributed to high levels of uncertainty about the illness, rising mortality rates [4], lack of effective cure, and risks to personal safety and their loved ones [8,9]. Emerging COVID-19 literature, as well as studies from previous pandemics, have highlighted the risk and negative impacts of moral distress and injury in health care [10,11] and social service providers [7].

Virtual education programs can be used to overcome barriers to healthcare and social service provider training in a pandemic situation arising from physical distancing, quarantine, and other isolation measures [12]. Extension for Community Healthcare Outcomes (ECHO) is a widely used virtual education model that has been implemented globally to build capacity and create virtual communities of practice [13,14]. The model helps to address issues related to complexities in care, disparities in access to care, and rapid diffusion of evidence-based practices [14]. It leverages videoconferencing technology to create a virtual community of practice, whereby primary care and other health care providers (“spokes”) connect with specialist teams (“hubs”) to collaborate, learn, and share best practices through regularly scheduled sessions. Each session typically consists of a brief didactic presentation on a relevant disease-related topic, followed by a spoke-provider presentation about an anonymized complex case of a patient in their care, and then a community discussion to consolidate learning and to work through the case to develop practical recommendations for the provider to take back to their practice [13,14].

Globally, organizations have adapted the ECHO model for their respective COVID-19–specific needs. A recent study [15] reported the use of the ECHO model in supporting health care providers mental well-being and resilience during the COVID-19 pandemic; however, use of the ECHO model to support social service providers and health care providers in caring for people with intellectual and developmental disabilities during the pandemic has yet to be reported.

COVID-19 was declared a pandemic on March 11, 2020. As a result of the pandemic, social service providers and health care providers who support people with intellectual and developmental disabilities faced new and unprecedented challenges in community settings. In response, we leveraged the ECHO model to develop ECHO Ontario Adult Intellectual and Developmental Disabilities: Mental Health in the Time of COVID-19 (ECHO AIDD-COVID), a targeted virtual education program to support care providers, working together, from the disability and health sectors. The purpose of the program was to share best practices in caring for the mental health of the intellectual and developmental disabilities population during the COVID-19 pandemic and to reduce feelings of isolation and burnout by making new connections and sharing resources. In this paper, we aim to describe the rapid development and evaluation of the ECHO AIDD-COVID program. We hypothesized that this program would improve participants’ self-efficacy in supporting and managing the mental health issues of people with intellectual and developmental disabilities during the COVID-19 pandemic.

## Methods

### General Design

The rampant increase in COVID-19 infections in the winter of 2020, and the subsequent need to enhance and strengthen the skills of care workers, led to the rapid planning and development of a COVID-19–focused ECHO program. This program was an adaptation of an existing 12-session ECHO program launched prior to the COVID-19 pandemic, ECHO Ontario Adult Intellectual and Developmental Disabilities (ECHO Ontario AIDD), which focused on caring for the mental health of people with intellectual and developmental disabilities [16].

A rapid design thinking approach [17] over 2 weeks, similar to plan-do-study-act cycles [18], guided our development process. Design thinking is a step-wise approach that involves observation, collaboration, fast learning, the visualization of ideas, rapid prototyping, feedback gathering, and redesign [19,20]. It is human-centered; incorporates creative problem-solving, co-design, low-fidelity prototyping; has an iterative design; and “bias towards action [19,20].” With the uncertainties related to illness outcomes experienced during the COVID-19 pandemic and ongoing rapidly emerging information, a flexible process that allowed for adjustments to be made quickly when developing and running programs was needed. Design thinking facilitated such adjustments and offers a structure for building creative and innovative solutions to complex problems that involve uncertainty [17]. The preferred approach has the added advantage of accelerated prototyping and testing; key steps in this approach included inspiration, ideation, and implementation [17].

### Stage 1: Inspiration

The inspiration stage—the problem or opportunity at hand—comprised the challenges created for providers caring for people with intellectual and developmental disabilities by the COVID-19 pandemic, such as the negative impact on mental health, for both clients and providers alike, and the need for rapid capacity building and connection in the community.

### Stage 2: Ideation

This stage involved brainstorming and refining ideas and solutions. We met several times with project leads, hub members, and members of the ECHO program team to explore ways to leverage existing operational structures and the collective expertise of the multidisciplinary ECHO Ontario AIDD team members.

### Stage 3: Implementation

In this stage, potential solutions were developed and shared with target users, who provided feedback. A prototype—a description used in rapid design framework to develop best possible solutions for the identified problems [17,21]—program was developed with COVID-19 pandemic–focused innovations within the existing structure and curriculum of the ECHO Ontario AIDD program. The innovative solutions in our prototype were (1) a curriculum that integrated COVID-19, mental health, and intellectual and developmental disabilities; (2) curated COVID-19 updates; (3) wellness checks and mindfulness sessions for self-care and wellness; and (4) a family member as one of the content experts in the hub. Content experts in the team had identified several unique COVID-19 mental health challenges faced by people with intellectual and developmental disabilities, and it was important to create opportunities that rapidly enhance the skills of care providers in the community. Moreover, the “infodemic” [22] of COVID-19 information highlighted the need to provide authentic, updated, and timely COVID-19 information to care providers. The specially designed curriculum and COVID-19 updates were creative solutions tailored for this purpose. Family members with lived experience, as content experts in the hub, brought an important perspective to the pedagogy of the program, specifically, by providing insight into how knowledge obtained from ECHO discussions could be translated and applied in the realistic care of clients. Early reports [23] from the pandemic highlighted the need to provide wellness tools to care providers to prevent burnout. We integrated wellness checks and mindfulness within the program to address this concern.

### ECHO AIDD-COVID Program

Participation in the ECHO AIDD-COVID program was open to all care providers, both social service providers and health care providers, working with people with intellectual and developmental disabilities in Ontario, Canada. We recruited potential participants by emailing invitation flyers to all previous participants of ECHO programs at the Centre for Addiction and Mental Health, as well to developmental service agencies, community mental health organizations, professional accrediting colleges, and primary care sites in Ontario. Providers who were interested in participating completed a web-based application form. All applicants independently providing care were accepted in to the program.

We assembled a hub team that comprised a psychiatrist, psychologist, primary care physician, behavior therapist, occupational therapist, nurse, social worker, and family advisor (the parent of an adult with intellectual and developmental disabilities). The strengths of the team included expertise in primary care, mental health, and intellectual and developmental disabilities; experience working directly with people with intellectual and developmental disabilities the during COVID-19; and experience managing psychological distress.

The curriculum was developed by triangulating sources, including feedback from a prior needs’ assessment [24], evaluation of the recently piloted ECHO Ontario AIDD cycle, review of COVID-19 literature on evidence-based practices to support health care providers, and consensus discussions within the hub members. Concerns related to the impacts of COVID-19 pandemic on people with intellectual and developmental disabilities were deterioration in mental health, worsening of challenging behaviors, overprescription of medications, and diagnostic overshadowing [25]. Hence, mental health was an important part of this program. Caregiver strain was identified as another area of need [26]. The final list of topics were COVID-19 overview (with relevance to intellectual and developmental disabilities); staff wellness and self-care; advanced care planning and supported health care decision-making during COVID-19; depression, anxiety, and evaluating risk; supporting families during COVID-19 and family interventions; and grief and loss. Because we did not know how long people would be willing and able to participate in an ECHO program during the pandemic, we opted for 6 sessions, which seemed sufficiently long to present key material and form a community of practice while also remaining efficient at spreading information.

Weekly 1.5-hour-long sessions were conducted from April 17, 2020 to May 22, 2020 over 6 weeks, which is half the duration of ECHO Ontario AIDD. Each session included introductions, a mindfulness exercise led by the family advisor, a wellness check, COVID-19–related research and policy updates, a didactic presentation based on the curriculum topic for the day, and a case-based discussion, in which a participant (care provider) presented an anonymized case from their practice, for which they required support, to illustrate the complexities in caring for people with intellectual and developmental disabilities in conjunction with the impact of COVID-19. Additionally, a web-based ECHO AIDD-COVID resource portal, with reference materials related to the ECHO program, was available to the participants for use during and after the sessions. Sessions had a dual focus— participant skill development, for addressing the mental health issues faced by people with intellectual and developmental disabilities, and support for the psychological well-being of participants. The innovations described earlier were integrated in to the ECHO sessions seamlessly. Evidence-based resources were shared with participants and could be accessed after the course.

### Evaluation

#### Overview

Our evaluation strategy was informed by the Evaluation framework for continuing professional development, specifically, levels 1 to 5 (participation, satisfaction, learning, self-efficacy and change in practice) [27]. This framework has been used globally to structure evaluations for ECHO [28-30]. We asked additional questions about participation in the program within the context of the COVID pandemic and reflections on innovations to the program.

#### Participation

Basic participant demographic information (profession, practice setting, and attendance) was collected throughout the duration of the program.

#### Satisfaction

Participant satisfaction was measured weekly using web-based postsession satisfaction questionnaires. Statements were rated on a 5-point Likert scale, from 1 (strongly disagree) to 5 (strongly agree), and focused on expanded knowledge and skills, reduced professional isolation, addressed learning needs, recommend session to others, and overall satisfaction. We obtained qualitative feedback with open-text responses to questions that asked for suggestions (for curriculum topics) and overall comments or feedback.

#### Learning and Competency

Perceived self-efficacy was assessed for 4 core program competencies, with respect to providing care for people with intellectual and developmental disabilities during the COVID-19 pandemic, before and after program participation, with a previously established 100-point confidence scale (a higher number indicated higher confidence) [28]. Competencies were developed by the ECHO AIDD-COVID hub team through team discussions on personal experience, review of the literature on care providers’ challenges in managing mental health care in the intellectual and developmental disabilities population during the pandemic, and expert consensus.

#### Change in Practice

Participants responded, using a binary scale (1, yes; 0, no), to a question that asked whether participation in the program resulted in a change in their practice; participants were also prompted to provide examples with open-text feedback.

#### Experiences With the COVID-19 Pandemic

Feedback in this area was collected before and after the program with 2 items—having professional support and being equipped to cope with stressors (ie, fear of contagion; rapid spread of virus; risk to self, client, family, or friends, etc) related to the pandemic—using a 5-point Likert scale from 1 (strongly disagree) to 5 (strongly agree).

Reflection questions about innovations to the program (combining social service providers and health care providers, COVID-19 strategy sharing, including of mindfulness practice, including support from the community of practice, and sharing COVID-19 updates) were asked after the program, with responses captured using a 5-point Likert scale. Participants were also asked to comment on how participation in the ECHO AIDD-COVID program impacted the challenges that they experienced during the COVID-19 pandemic with open-text responses.

Evaluation measures and data sets generated and analyzed in this report were reviewed and deemed to be part of program evaluation at the Centre for Addiction and Mental Health.

### Data Analysis

Quantitative data were analyzed using either Excel (Microsoft Inc) or SPSS software (version 21; IBM Corp). Proportions, frequencies, and percentages were calculated for categorical variables (profession, practice setting, change in practice, and attendance). Means and standard deviations were calculated for continuous variables (satisfaction scores, self-efficacy scores, scores for reflections about innovations to the program, and scores for experiences with COVID-19 pandemic). Pre- and postprogram data about experiences during the COVID-19 pandemic and self-efficacy were analyzed using either paired *t* tests or Wilcoxon signed-rank tests, as appropriate. Statistical tests were 2-sided, with a statistical significance level of 5%.

Preliminary modified manifest content analysis was conducted using NVivo software (version 12, QSR International) to evaluate open-text responses about program participation and the impact to challenges experienced during COVID-19. A project team member uploaded all open-text responses into NVivo, and then reviewed and performed open coding on all text. The project team met on a regular basis to review and discuss coding to develop and refine a coding matrix with definitions. This coding matrix was applied to all text; references for each code were reviewed, and frequencies were calculated. Finally, all codes were summarized and organized for interpretation [31,32].

## Results

A total of 104 care providers, with a variety of professional backgrounds, from 56 organizations (Table 1) attended 1 or more sessions (participants per session: mean 81), and more than 88% of participants (92/104) attended at least half of the sessions.

Weekly satisfaction scores were high, ranging from a mean 4.07 (SD 0.18) to a mean 4.32 (SD 0.14); the overall mean satisfaction score was 4.31 (SD 0.17; Table 2).

**Table 1:** Participant demographics.

Demographic group			Value, n (%)
**Participants by profession**			104 (100)
	Access coordinator or service navigator	2 (1.9)	
	Administrator	5 (4.8)	
	Behavior analyst	6 (5.8)	
	Case worker or manager	23 (22.1)	
	Developmental services professional	16 (15.4)	
	Physician	8 (7.7)	
	Nursing professional (registered nurse, registered practical nurse, nurse practitioner)	8 (7.7)	
	Occupational therapist	2 (1.9)	
	Other (pharmacist, speech language pathologist)	4 (3.8)	
	Psychologist or psychotherapist	2 (1.9)	
	Social worker	19 (18.3)	
	Support worker	9 (8.7)	
**Organizations by practice setting**			56 (100)
	Academic hospital	3 (5)	
	Community health center	3 (5)	
	Community mental health agency	6 (11)	
	Community mental health and addictions agency	1 (2)	
	Community support services agency	10 (18)	
	Developmental services community agency	28 (50)	
	Family health group	1 (2)	
	Family health team	1 (2)	
	Other	1 (2)	
	Private practice or solo practitioner	2 (4)	

**Table 2:** Ratings for satisfaction survey items.

Item	Rating out of 5 (n=228)^a^, mean (SD)
The session content expanded my existing skills and knowledge.	4.07 (0.18)
This session has addressed my learning needs.	4.15 (0.07)
This session has reduced my professional isolation.	4.21 (0.18)
I would recommend this session to others.	4.32 (0.14 )
Overall, I was satisfied with the session.	4.31 (0.17)

^a^Total number of completed weekly surveys received.

In total, 42 participants completed both pre- and postprogram self-efficacy and experiences with the COVID-19 pandemic questionnaires. Mean self-efficacy scores prior to participation in ECHO were 61.3 (SD 18.2), and after the program, self-efficacy scores were 81.1 (SD 9.8); there was a statistically significant improvement (t_41_= –9.035, *P*<.001; *d*=1.394). Analysis of individual statements (core program competencies) also showed statistically significant improvements in mean self-efficacy scores (all *P*<.001). Statistically significant differences were also seen for pre-ECHO to post-ECHO mean scores for both statements on experiences with COVID-19 (ie, professional support and being equipped to cope with stressors related to the pandemic). Table 3 shows mean self-efficacy scores for individual core program competency statements and mean scores for experience with COVID-19 items.

**Table 3:** Change in change in pre- and postprogram confidence and experience with COVID-19 participation in the ECHO AIDD-COVID program.

Items		Score (n=42), mean (SD)				*P* value
		Pre	Post	Difference		
**Core program competencies**						
	Communicate effectively and prepare for person and family-centered care for adults with intellectual and developmental disabilities during the COVID-19 pandemic	65.38 (20.22)	81.83 (9.79)	16.45 (17.72)	<.001^a^	
	Support and manage the mental health of individuals with or suspected of having intellectual and developmental disabilities during the COVID-19 pandemic	56.47 (20.06)	77.88 (13.54)	21.40 (16.39)	<.001^b^	
	Manage burnout and build resilience in myself, other health care and developmental service professionals, and caregivers during the COVID-19 pandemic	57.57 (22.34)	78.52 (12.08)	20.95 (21.44)	<.001^c^	
	Work effectively in/with interprofessional and intraprofessional teams across health and social systems during the COVID-19 pandemic to support the care of clients with intellectual and developmental disabilities	69.33 (18.04)	86.07 (10.09)	16.74 (15.72)	<.001^d^	
**COVID-19 experience**						
	I feel I have enough professional support and resources for myself to continue caring for my clients during this time	3.45 (0.89)	4.10 (0.62)	0.64. (0.82)	<.001^e^	
	I feel equipped to cope with stressors (ie fear of contagion, rapid spread of virus, risk to self/client/family/friends, etc) related to the COVID-19 pandemic	3.17 (0.93)	4.10 (0.66)	0.93 (0.87)	<.001^f^	

^a^The Wilcoxon signed rank test was used (*Z*= –4.728; *r*=0.730).

^b^A paired *t* test was used (t_41_= –8.464; *d*=1.306).

^c^A paired *t* test was used (t_41_= –6.335; *d*=0.977).

^d^A paired *t* test was used (t_41_= –6.902; *d*=1.065).

^e^A paired *t* test was used (t_41_= –5.074; *d*=0.783).

^f^A paired *t* test was used (t_41_= –6.945; *d*=1.072).

The analysis of 53 open-text responses about the impact of ECHO participation on challenges experienced by participants during the COVID-19 pandemic is summarized in Table 4. Key areas that emerged within the responses included: ECHO expanding participants’ knowledge and competency (29/53, 55%); being part of a community of practice in ECHO (25/53, 47%); reflections on the value of the COVID-19 innovations (21/53, 40%); ECHO supporting the gaining and sharing of resources, not just within the smaller ECHO community, but also with participants’ teams and organizations (18/53, 34%); ECHO facilitating changes to participants’ practice via their approach to client care (10/53, 19%); and feelings of increased confidence in supporting clients and families (3/53, 6%).

**Table 4:** Key areas that emerged from open-text responses.

Key areas			Participants (n=53), n (%)^a^
**Expanding knowledge and competency**			29 (55)
	Benefits of case-based learning	9	
	Improvements to knowledge and awareness	13	
	Increased learning through interprofessional education	5	
**Being part of a community of practice**			25 (47)
	Supporting and learning from one another	9	
	Validation from others	2	
**Reflections on COVID-19 innovations**			21 (40)
	Benefits of mindfulness	4	
	Increases in COVID-19 knowledge	12	
	Value of family perspective	5	
**Gaining and sharing resources**			18 (34)
	Sharing resources with broader teams and organizations	3	
**Facilitating changes to practice**			10 (19)
	Application of knowledge in client care	7	
Increased confidence in supporting clients and families			3 (6)

^a^Percentages do not add to 100%, and only n values are provided for subthemes.

Participants were also asked about participation in ECHO having an impact on practice. Participants reported participation in ECHO AIDD-COVID resulted in a change in their practice and an equal number were in favor of this program being run again (46/53, 87%). Almost all of the participants reported their learning needs were met in the program (51/53, 96%). The reflection questionnaire around prototype innovations in the program was completed by 53 participants. An overwhelming 98% of participants (52/53) agreed ECHO AIDD-COVID made them feel supported and part of a virtual community of practice. One participant commented that it was

so nice to know that we are not alone in this strange time and share the same challenges

and that they would

miss this weekly touch point with professionals.

A similar percentage of participants (52/53, 98%) reported COVID-19 updates and resources as valuable; a participant shared,

there were innovative strategies suggested in each session as well as content in the presentations that I believe helped improve my approach day to day with the clients I have been supporting.

Furthermore, 94% of participants (50/53) agreed or strongly agreed that having both interprofessional health care providers and social service providers enhanced their learning. Most participants (51/53, 96%) also reported that having a family member in the hub enhanced their learning. The impact of the family perspective is best illustrated by a participant who shared that

...the most powerful experiences I had was whenever the family member spoke. I think we all can hypothetically understand caregivers’ perspectives, but we cannot understand the full emotional toll or the personal thoughts and worries that caregivers have.

A similar percentage (51/53, 96%) of participants appreciated the opportunity to share strategies in the community. The weekly mindfulness exercise led by the family advisor was reported to be helpful by 77% of the participants (41/53) and functioned to

remind us to take care of ourselves.

## Discussion

### General

We described the successful development and evaluation of a COVID-19–focused ECHO program for workers caring for people with intellectual and developmental disabilities. A rapid design thinking approach was used to develop the ECHO AIDD-COVID program. Evaluation findings showed high levels of engagement and satisfaction with the program, with the majority of participants reporting changing their practice because of the program. To the best of our knowledge, this paper is the first to document use of the ECHO model and its significant improvement in perceived self-efficacy in caring for people with intellectual and developmental disabilities during the COVID-19 pandemic for a cohort that encompassed both social service providers and health care providers. Improved confidence in all the core program competencies shows the ECHO model is an effective way to improve provider skills for supporting the mental health needs of people with intellectual and developmental disabilities. Additionally, there was a cascading effect from knowledge shared by participants beyond the program—use of the program can be an effective share-and-spread strategy during the pandemic. Participant feedback from open-text responses shows that the program helped expand knowledge and facilitated changes in practice. The ECHO model was conceptually designed to develop a community of practice. This was validated by the qualitative feedback from participants. The results of the pre- and postprogram evaluations suggest that participants felt that group participation helped to support their own well-being, especially their ability to cope with COVID-19 stressors.

### Rapid Design Thinking and COVID-19

Because of the pandemic, uncertainty and unprecedented challenges arose in social support and health care sectors, and there was a need for the swift deployment of a capacity-building program to support the needs of people with intellectual and developmental disabilities and those of the health and social service providers who work with this underserved population. The rapid design thinking framework was instrumental in incorporating lessons learned from our previous ECHO capacity-building project [16], to adapt to COVID-19–specific needs, develop a purpose-built prototype, and implement the design within a short timeframe. The design thinking principles are helpful in volatile, uncertain, complex, and ambiguous situations [33], and the COVID-19 pandemic presents such conditions. Additionally, there is evidence in medical education literature that supports the use of design thinking [21,34,35]. Other agile methods [36] that are similar to rapid design thinking can be explored in developing similar programs. The attendance rate, high levels of satisfaction, and retention of the program suggest the acceptability of the shortened 6-session program. Interestingly, retention rates were similar to those of the prior 12-week ECHO program targeted toward the same audience. It may be that in a time of intense duress, people are able to commit to a short intervention, with enough support being conveyed over this short period to make a difference. Additional research is necessary to determine if a brief program produces as much change as that from a longer program and whether attendance is better in one program or the other. Rapid prototyping and testing were key aspects of the program; we utilized qualitative and quantitative findings from the previous iteration and introduced several time-sensitive innovations including COVID-19–specific content, such as advance care planning and care provider wellness. The learn-as-you-go aspect of the rapid design thinking framework was evident in the iterative nature of the model [17,34], guided by feedback obtained from weekly satisfaction surveys.

### Innovations That Made a Difference

In contrast to other ECHOs designed to strictly focus on working with a population [37] or addressing worker mental health [15], this course integrated both aspects. High levels of satisfaction with the innovations in the COVID-19 prototype program may have contributed to its success. Participants appreciated the COVID-19–specific content of the program, and almost all questionnaire respondents indicated an intent to apply learning in practice. Curated COVID-19 information and weekly updates, self-care tips, and family perspectives were prominent in participants’ reflections on COVID-19–related innovations in the program. Several participants spoke about the mindfulness exercises and how they helped them to be more aware of their own wellness needs during the pandemic. We recognized that, while it is important to provide practical clinical tools to providers, it is not enough to address clinical needs without recognizing the impacts of moral distress and injury on frontline care providers [7]. The wellness checks and community mindfulness exercises were opportunities to share ideas about coping during the pandemic. Participants also reflected on the program’s contribution as a virtual community of practice. “*Feeling connected*” and “*we are not alone*” came out strongly in the reflections, which were indicators of the program bringing professionals from disability and health services sectors together as a community. Sockalingam et al [15] concluded that virtual communities of practice that focus on self-care skills development and support for frontline health care workers are needed to address emerging distress, fatigue, and mental health needs during this pandemic. The curriculum was coproduced and co-delivered with a family advisor hub member. Similar to descriptions in literature [38-40], the family-centered care perspective was deeply valued by participants. Training in partnership with patients and providers helps in developing empathy and desired professional attitudes [40]. At a pedagogical level, this highlights the important role of caregivers as educators and working in partnership to improve provision of care [41,42].

### Limitations

There are some limitations to consider when reviewing our findings. First, the data and measures used in this evaluation are from a single cycle of an ECHO program that was specifically focused on mental health in intellectual and developmental disabilities populations in Ontario during COVID-19; as such, our findings may not be generalizable to other cycles or settings. Future evaluation and research efforts will seek to replicate these findings with other health conditions and settings. Additionally, findings for satisfaction, changes in confidence, and experience with COVID-19 participation were informed by data collected from individuals who completed satisfaction surveys and questionnaires. This may introduce a response bias, whereby those who participated in these data collection activities may have been more engaged and likely to respond with higher scores; however, we recognize this challenge is not unique to our evaluation and exists for anyone collecting data via surveys.

### Future Directions

Future iterations should incorporate targeted outcome measures to evaluate the role of ECHO in addressing the mental health needs of care workers who are supporting people with intellectual and developmental disabilities in the community [15]. The virtual, synchronous nature of the ECHO model was valuable during the pandemic, as the timing and outreach of the capacity-building program was essential, and travel restrictions were major impediments to any in-person training. Future work should also include the perspective of adults with intellectual and developmental disabilities in teaching. Emerging literature has highlighted the importance of people with intellectual and developmental disabilities in medical education [43,44].The role of *expert patients* as educators in the ECHO model needs further exploration.

Although this study addressed self-efficacy and the competency of learners, focus on retention and its medium- to long-term effects would be helpful for designing future programs. In addition, the evaluation of implementation outcomes would be helpful to understand the impact of this educational intervention. Ethical implications and dynamics involved in coproducing educational content are important future considerations [42]. We will continue with this program at a provincial and national level as long as Canada continues to face COVID-19–related restrictions, and we plan to measure long-term outcomes such as staff burnout and COVID-19–related stress in future iterations. Furthermore, by scaling up capacity-building initiatives nationally, a wider community of practice can be built, which has implications for knowledge translation research at a systems level.

## Abbreviations

ECHO: Extension for Community Healthcare Outcomes
ECHO AIDD-COVID: Extension for Community Healthcare Outcomes Ontario Adult Intellectual and Developmental Disabilities: Mental Health in the Time of COVID-19
ECHO Ontario AIDD: ECHO Ontario Adult Intellectual and Developmental Disabilities

## References

[1] Courtenay K, Perera B (2020). COVID-19 and people with intellectual disability: impacts of a pandemic. Ir J Psychol Med.

[2] Constantino JN, Sahin M, Piven J, Rodgers R, Tschida J (2020). The impact of COVID-19 on individuals with intellectual and developmental disabilities: clinical and scientific priorities. Am J Psychiatry.

[3] Perera B, Laugharne R, Henley W, Zabel A, Lamb K, Branford D, Courtanay K, Alexander R, Purandare K, Wijeratne A, Radhakrishnan V, McNamara E, Daureeawoo Y, Sawhney I, Scheepers M, Taylor G, Shankar R (2020). COVID-19 deaths in people with intellectual disability in the UK and Ireland: descriptive study. BJPsych Open.

[4] Clift A, Coupland C, Keogh R, Hemingway H, Hippisley-Cox J (2021). COVID-19 mortality risk in Down syndrome: results from a cohort study of 8 million adults. Ann Intern Med.

[5] Callea M, Cammarata-Scalisi F, Galeotti A, Villani A, Valentini D (2020). COVID-19 and Down syndrome. Acta Paediatr.

[6] Sheehan R, Dalton-Locke C, Ali A, Vera San Juan Norha, Totsika Vaso, Hassiotis A (2021). Effects of the COVID-19 pandemic on mental healthcare and services: results of a UK survey of front-line staff working with people with intellectual disability and/or autism. BJPsych Bull.

[7] Bobbette N, Hamdani Y, Thomson K, Abou-Chacra M, Volpe T, Lunsky Y Recognizing the mental health needs of an essential workforce: being a direct support professional in the time of COVID-19. Centre for Addiction and Mental Health Azrieli Adult Neurodevelopmental Centre.

[8] Kisely S, Warren N, McMahon L, Dalais C, Henry I, Siskind D (2020). Occurrence, prevention, and management of the psychological effects of emerging virus outbreaks on healthcare workers: rapid review and meta-analysis. BMJ.

[9] Preti E, Di Mattei V, Perego G, Ferrari F, Mazzetti M, Taranto P, Di Pierro R, Madeddu F, Calati R (2020). The psychological impact of epidemic and pandemic outbreaks on healthcare workers: rapid review of the evidence. Curr Psychiatry Rep.

[10] Williamson V, Murphy D, Greenberg N (2020). COVID-19 and experiences of moral injury in front-line key workers. Occup Med (Lond).

[11] Greenberg N, Docherty M, Gnanapragasam S, Wessely S (2020). Managing mental health challenges faced by healthcare workers during covid-19 pandemic. BMJ.

[12] Torous J, Jän Myrick Keris, Rauseo-Ricupero N, Firth J (2020). Digital mental health and COVID-19: using technology today to accelerate the curve on access and quality tomorrow. JMIR Ment Health.

[13] Struminger B, Arora S, Zalud-Cerrato S, Lowrance D, Ellerbrock T (2017). Building virtual communities of practice for health. Lancet.

[14] Arora S, Kalishman SG, Thornton KA, Komaromy MS, Katzman JG, Struminger BB, Rayburn WF, Bradford AM (2017). Project ECHO: a telementoring network model for continuing professional development. J Contin Educ Health Prof.

[15] Sockalingam S, Clarkin C, Serhal E, Pereira C, Crawford A (2020). Responding to health care professionals' mental health needs during COVID-19 through the rapid implementation of project ECHO. J Contin Educ Health Prof.

[16] Bobbette N, Hardy J, Hamdani Y, Serhal E, Sockalingam S, Lunsky Y (2020). The development and evaluation of a pilot cross sector Project ECHO program focused on the mental health of adults with intellectual and developmental disabilities.

[17] Thakur A, Soklaridis S, Crawford A, Mulsant B, Sockalingam S (2021). Using rapid design thinking to overcome COVID-19 challenges in medical education. Acad Med.

[18] Taylor Michael J, McNicholas Chris, Nicolay Chris, Darzi Ara, Bell Derek, Reed Julie E (2014). Systematic review of the application of the plan-do-study-act method to improve quality in healthcare. BMJ Qual Saf.

[19] Abookire S, Plover C, Frasso R, Ku B (2020). Health design thinking: an innovative approach in public health to defining problems and finding solutions. Front Public Health.

[20] Lockwood T (2010). Design Thinking: Integrating Innovation, Customer Experience and Brand Value.

[21] Deitte LA, Omary RA (2019). The power of design thinking in medical education. Acad Radiol.

[22] Rutter H, Wolpert M, Greenhalgh T (2020). Managing uncertainty in the covid-19 era. BMJ.

[23] Bertuzzi Vanessa, Semonella Michelle, Bruno Denise, Manna Chiara, Edbrook-Childs Julian, Giusti Emanuele M, Castelnuovo Gianluca, Pietrabissa Giada (2021). Psychological support interventions for healthcare providers and informal caregivers during the COVID-19 pandemic: a systematic review of the literature. Int J Environ Res Public Health.

[24] Vogt J, Huijbregts M, Isaacs B (2020). COVID-19: Toronto Developmental Services Alliance Community Needs Assessment Agency survey responses: April 5-22, 2020. Real Xchange.

[25] Tromans S, Kinney M, Chester V, Alexander R, Roy A, Sander JW, Dudson H, Shankar R (2020). Priority concerns for people with intellectual and developmental disabilities during the COVID-19 pandemic. BJPsych Open.

[26] Willner Paul, Rose John, Stenfert Kroese Biza, Murphy Glynis H, Langdon Peter E, Clifford Claire, Hutchings Hayley, Watkins Alan, Hiles Steve, Cooper Vivien (2020). Effect of the COVID-19 pandemic on the mental health of carers of people with intellectual disabilities. J Appl Res Intellect Disabil.

[27] Moore DJ, Green J, Gallis H (2009). Achieving desired results and improved outcomes: integrating planning and assessment throughout learning activities. J Contin Educ Health Prof.

[28] Sockalingam S, Arena A, Serhal E, Mohri L, Alloo J, Crawford A (2018). Building provincial mental health capacity in primary care: an evaluation of a project ECHO mental health program. Acad Psychiatry.

[29] Zhou C, Crawford A, Serhal E, Kurdyak P, Sockalingam S (2016). The impact of project ECHO on participant and patient outcomes: a systematic review. Acad Med.

[30] McBain RK, Sousa JL, Rose AJ, Baxi SM, Faherty LJ, Taplin C, Chappel A, Fischer SH (2019). Impact of project ECHO models of medical tele-education: a systematic review. J Gen Intern Med.

[31] Hsieh H, Shannon SE (2005). Three approaches to qualitative content analysis. Qual Health Res.

[32] Erlingsson C, Brysiewicz P (2017). A hands-on guide to doing content analysis. Afr J Emerg Med.

[33] Cousins B (2018). Design thinking: organizational learning in VUCA environments. Academy Strateg Manage J.

[34] Gottlieb M, Wagner E, Wagner A, Chan T (2017). Applying design thinking principles to curricular development in medical education. AEM Educ Train.

[35] Roberts JP, Fisher TR, Trowbridge MJ, Bent C (2016). A design thinking framework for healthcare management and innovation. Healthc (Amst).

[36] Kushniruk Andre W, Borycki Elizabeth M (2015). Integrating low-cost rapid usability testing into agile system development of healthcare IT: a methodological perspective. Stud Health Technol Inform.

[37] Chopra N, Pereira C, Prata A, Lamba W, Sockalingam S (2021). Building substance use disorder management capacity during COVID-19: outcomes from a tele-mentoring program for community-based healthcare professionals. Can J Addict.

[38] Parham D, Reed D, Olicker A, Parrill F, Sharma J, Brunkhorst J, Noel-MacDonnell J, Voos K (2019). Families as educators: a family-centered approach to teaching communication skills to neonatology fellows. J Perinatol.

[39] Johnson AM, Yoder J, Richardson-Nassif K (2006). Using families as faculty in teaching medical students family-centered care: what are students learning?. Teach Learn Med.

[40] Biswas AB, Raju LB, Gravestock S (2018). Training in partnership: role of service users with intellectual disability and carers. Psychiatr Bull.

[41] Gordon M, Gupta S, Thornton D, Reid M, Mallen E, Melling A (2020). Patient/service user involvement in medical education: a best evidence medical education (BEME) systematic review: BEME Guide No. 58. Med Teach.

[42] Soklaridis S, de Bie A, Cooper RB, McCullough K, McGovern B, Beder M, Bellisimo G, Gordon T, Berkhout S, Fefergrad M, Johnson A, Kalocsai C, Kidd S, McNaughton N, Ringsted C, Wiljer D, Agrawal S (2020). Co-producing psychiatric education with service user educators: a collective autobiographical case study of the meaning, ethics, and importance of payment. Acad Psychiatry.

[43] Thomas B, Courtenay K, Hassiotis A, Strydom A, Rantell K (2014). Standardised patients with intellectual disabilities in training tomorrow's doctors. Psychiatr Bull.

[44] Trollor JN, Ruffell B, Tracy J, Torr JJ, Durvasula S, Iacono T, Eagleson C, Lennox N (2016). Intellectual disability health content within medical curriculum: an audit of what our future doctors are taught. BMC Med Educ.

